# Oral Administration of Shark Type II Collagen Suppresses Complete Freund’s Adjuvant-Induced Rheumatoid Arthritis in Rats

**DOI:** 10.3390/ph5040339

**Published:** 2012-03-28

**Authors:** Lijuan Chen, Bin Bao, Nanping Wang, Jing Xie, Wenhui Wu

**Affiliations:** 1 Shanghai Ocean University, No. 999, Hu Cheng Loop-road, Lingang New City, Shanghai 201306, China; Email: Clijuan_05shipin@163.com (L.C.); 2 Shanghai Fisheries Research Institute, No. 265 Jiamusi Road, Yangpu District, Shanghai 200433, China; Email: yujiash@hotmail.com

**Keywords:** shark type II collagen, rheumatoid arthritis, oral tolerance

## Abstract

*Objective*: Shark type II collagen (SCII) is extracted as a glycoprotein from the cartilage of blue shark *(Prionace glauca*). We aim to confirm the effects of oral tolerance of SCII on inflammatory and immune responses to the ankle joint of rheumatoid-arthritis rats induced by Complete Freund’s Adjuvant (CFA). *Materials and Methods*: The onset of rheumatoid arthritis (RA) was observed 14 ± x days after injection of CFA. Rats in the control group were treated with acetic acid by oral administration (0.05 mmol kg^−1^d^−1^, days 14–28), while rats in experimental groups were treated by oral administration with SCII (1 or 3 mg kg^−1^d^−1^, days 14–28), *Tripterygium wilfordii* polyglycosidium (TWP) (10 mg kg^−1^d^−1^, days 14–28), and bovine type II collagen from US (US-CII) (1 mg kg^−1^d^−1^, days 14–28), respectively. The severity of arthritis was evaluated by the articular swelling. The immunological indexes observed included delayed type hypersensitivity (DTH) reaction, the level of interleukins 10 (IL-10) in rat blood serum and morphological characterization. Mixed lymphocyte culture (MLC) was performed to investigate the relationship between T cell apoptosis and specific immune tolerance induced by SCII. *Results*: Treatment with SCII for 2 weeks significantly attenuated the acute inflammation. The rats orally administrated with SCII at the level of 3 mg kg^−1^d^−1^ (SCII 3) and US-CII had decreased DTH reaction compared with rats in control group. Rats treated with SCII 3 had the highest level of IL-10 with 102 pg/mL. SCII with concentration of 10 μg/L could help to significantly enhance level of Fas/Apo-1 in T cell *in vitro*. The result of histological staining indicated that the recovery of the articular membranes of ankle joint in SCII 3 group was greatly enhanced. *Conclusions*: Our results suggest that appropriate dose of SCII can not only ameliorate symptoms but also modify the disease process of Complete-Freunds-Adjuvant-induced arthritis. Oral administration of SCII might be a potential candidate as a novel drug for further investigation.

## 1. Introduction

Autoimmunity is an acquired immune reactivity to self antigens. Autoimmune diseases occur when autoimmune responses lead to tissue damage [1]. Rheumatoid arthritis (RA) is a chronic inflammatory autoimmune disease, which is characterized by chronic inflammation of the synovial tissues in multiple joints. RA can lead to joint destruction through inflammatory involvement of the synovial membrane, cartilage, and subchondral bone. In clinical, there are various drugs that are used as traditional treatment for symptomatic relief, such as azathioprine [2], minocycline, sulfasalazine [3], tumor necrosis factor alpha (TNFα) blockers, non-steroidal anti-inflammatory drug (NSAIDs) [4], *etc*. Although the drugs can temporarily alleviate the symptoms, they do not halt progression of joint destruction and they are accompanied with many undesirable adverse effects [5]. 

Inflammatory autoimmune diseases can be regarded as a failure of the immune system to maintain tolerance to certain self determinants, thus oral tolerance has been proposed for treating RA [6]. Oral tolerance is a key feature of the intestinal immunity, generating no responsiveness to ingested antigens [7]. This is a form of peripheral immune tolerance in which mature lymphocytes in the peripheral lymphoid tissues are rendered non functional or hyporesponsive by prior oral administration of antigen. The effects of oral administration of collagen, a plausible auto-antigen for RA, have been evaluated in various models of arthritis, among which orally administered type II collagen (CII) from bovine, chicken and human can suppress collagen-induced arthritis (CIA) and adjuvant-induced arthritis (AA) [8,9,10,11,12]. Moreover, CII has been used in the treatment of RA patients. Patients with RA disease participated to a placebo-controlled study or a multicenter, double blind, placebo-controlled trial, and a significant improvement of clinical indexes of disease activity (swollen and painful joint scores) was observed in the chicken CII treated group [13,14]. Oral administration of type II collagen from land animals is favor of the recovery of RA disease by introducing antigen-specific tolerance. However, the mechanisms how oral administration of CII to reduce and repair the damaged joint are still unclear.

Type II collagen is the principal component of extracellular matrix of articular cartilage. It is composed of 15–25% of the wet weight, about half the dry weight and 90–95% of the total collagen content in the cartilage [15]. In current market, most type II collagen is traditionally extracted from bovine articular cartilage, porcine articular cartilage, and chick sternal cartilage. However, the application of the type II collagen from traditional source are usually complicated by outbreak of bovine spongiform encephalopathy (BSE) crisis, transmissible spongiform encephalopathy (TSE) crisis and the foot-and-mouth disease (FMD) crisis. People now have taken more interest in marine collagen from fish scale, skin and bone, which are sold as supplements and cosmetics in the market. Shark cartilage is commonly used as dietary supplement in the form of powder to prevent a variety of illnesses, notably cancer. Moreover, shark skin collagen has been reported as an important source of marine collagen [16]. 

In order to develop new therapeutic agents that can prevent damage of arthritic joints with fewer adverse effects and lower cost, the present study was carried out by orally administering CII extracted from shark to adjuvant-induced arthritis rats. A RA animal model was built to investigate the effect of oral tolerance of shark CII (SCII) on RA. *Tripterygium wilfordii* polyglycosidium (TWP) from a drug store and pure bovine type II collagen made in the United Stated (US-CII) were used as positive control drugs. Investigation of tolerance in T cell induced by SCII *in vitro* further suggests the underneath mechanism how type II collagen by oral tolerance reduce and repair the damaged joint.

## 2. Results

### 2.1. Peptide Mapping

The subunit compositions of shark CII (SCII) from shark cartilage were examined by SDS-PAGE using separate gels of 7.5% polyacrylamide to directly compare the pattern of a middle molecular weight marker. According to the principle of SDS-PAGE, collagen subspecies band would typically appeared in a characteristic ladder-like array in their SDS-PAGE patterns. 

**Figure 1:**
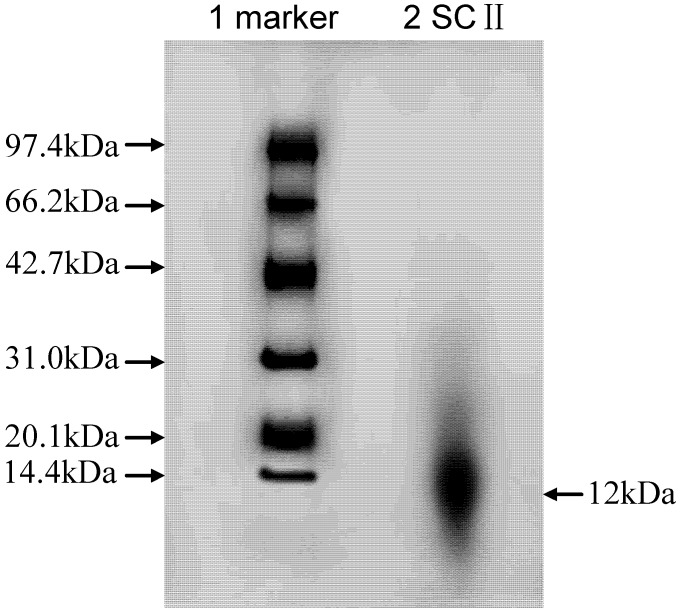
SDS-polyacrylamide gel electrophosesis pattern of collagen from shark cartilage. Electrophoresis was performed on 7.5% polyacrylamide gel and the peptides were stained with Coomassie Brilliant Blue. A 12 kDa peptide, corresponding to the predicated size of molecular weight protein marker, was present in the sample band. Line 1: molecular weight protein marker; Line 2: type II collagen from shark cartilage.

As shown in Figure 1, similar results were observed in our study, which showed that SCII was essentially free from contaminating proteins and the molecular of its subspecies was near 12 kDa.

### 2.2. Clinical Assessment

All animals tolerated the experimental procedures well with no evidence of drug toxicity and no deaths up to the study termination at Day 28. The onset and distribution of arthritis were similar to the pattern previously described [5]. Complete Freund’s Adjuvant (CFA) groups were induced by injecting CFA intradermally into rats on day 1. Ankle joint swelling began to develop on the 3rd day after CFA injection. On Day 14, the joint thickness of each rat, the scores for the paws, and the body weight of each group after their starvation for 8 h were assessed as given in Table 1. From the table, we can see that all CFA groups were successfully induced rheumatoid arthritis in 100% (10 of 10) of the injected Wister rats. A RA animal model used to evaluate the effect of drugs for treatment was successfully built up.

**Table 1:** The clinical manifestations in 2 weeks for each experiment group after injecting CFA or acetic acid.

Group	Blank	Control	US-CII	SCII1	SCII3	TWP
Body weight(g)	160 ± 11	169 ± 24	166 ± 15	151 ± 16	176 ± 14	167 ± 17
scores	0.0	2.8	2.7	2.8	2.8	3.0
Diameter of right ankle joint (mm)	7.92 ± 0.29	7.94 ± 0.43	7.78 ± 0.43	7.86 ± 0.53	7.81 ± 0.21	7.47 ± 0.30
Diameter of left ankle joint (mm)	8.07 ± 0.71	8.62 ± 0.46 **	8.35 ± 0.38 **	8.58 ± 0.66 **	8.61 ± 0.29 **	8.38 ± 0.42 **

All experiment groups except for the blank group were injected the CFA in the left hind paw on the 1st day to induce RA. The CFA-induced-rheumatoid-arthritis rat model was set up after 2 weeks. ** *P* < 0.01, statistically significantly different from its diameter of right ankle joint.

### 2.3. Appearance Characteristic of Ankle

To evaluate the clinical effect of SCII on CFA-induced arthritis, the clinical indices of arthritis were measured on the 28th day of study. The results obtained were shown in Table 2. The control group had the highest arthritis score up to 3.4 in the CFA groups; while the scores of other CFA groups were lower, suggesting that inflammation of arthritis in US-CII group, SC II groups and TWP group was attenuated via oral administration of the above drugs. Articular swelling was reduced after administration for 2 weeks, with the diameter changes of left ankle joint from 8.35 ± 0.38 mm to 8.25 ± 0.39 mm, 8.58 ± 0.66 mm to 8.52 ± 0.39 mm, 8.61 ± 0.29 mm to 8.52 ± 0.38 mm, 8.38 ± 0.42 mm to 8.16 ± 0.24 mm in US-CII group, SCII 1 groups (oral SCII of 1 mg kg^−1^d^−1^), SC II 3 groups (oral SCII of 3 mg kg^−1^d^−1^) and TWP group, respectively. The diameter of left ankle joint in blank group kept the same level as time went on. However, the left ankle joint in control group continued to swell without any trend of recovery.

**Figure 2:**
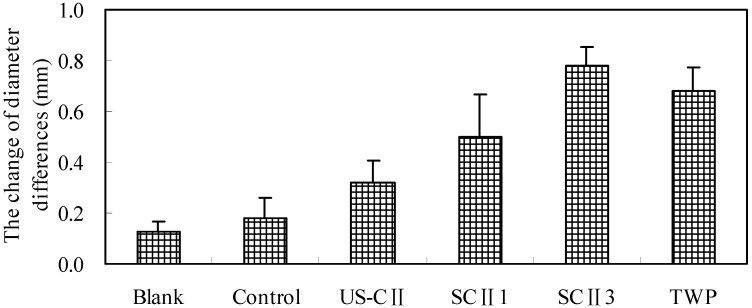
The change of diameter differences between left and right ankle joint from Day 14 to Day 28 (n = 10/group): diameter difference between the right ankle joint and the left ankle joint on Day 28 was subtracted from diameter difference between the right ankle joint and the left joint on Day 14, the values of blank group, control group, US-CII group, SCII1 group, SCII3 group and TWP group were 0.13 mm, 0.18 mm, 0.32 mm, 0.50 mm, 0.78 mm and 0.67 mm, respectively.

The recovery effects of CII and TWP were assessed by the change of diameters discrepancy between left and right ankle joint from Day 14 to Day 28, as shown in Figure 2. The change in control group (0.11 mm) was the smallest comparing to that in CII groups and TWP group. The greatest variation was observed in SCII 3 group (0.78 mm), indicating that administering SCII with the dose of 3 mg kg^−1^d^−1^ had the best effect of recovery for RA disease.

**Table 2:** The clinical manifestations for each experiment group after keeping administrating corresponding drug for 2 weeks.

Group	Blank	Control	US-CII	SCII1	SCII3	TWP
Body weight (g)	340 ± 10	288 ± 39	271 ± 26	245 ± 35	282 ± 13	284 ± 29
Scores	0.0	3.4	1.3	1.3	1.2	1.2
Diameter of right ankle joint (mm)	8.08 ± 0.52	8.53 ± 0.44	8.00 ± 0.41	8.30 ± 0.43	8.50 ± 0.38	7.92 ± 0.23
Diameter of left ankle joint (mm)	8.10 ± 0.39	9.03 ± 0.90 *	8.25 ± 0.39 **	8.52 ± 0.39 **	8.52 ± 0.38 **	8.16 ± 0.24 **

All drugs were dissolved in 0.05M acetic acid and orally administered to rat from 14 days to the treatment of CFA; Values represent the mean ± SEM of 10 animals for each group. * *P* < 0.05, statistically significantly different from its diameter of right ankle joint. ** *P* < 0.01, statistically significantly different from its diameter of right ankle joint.

### 2.4. DTH Responses

**Figure 3:**
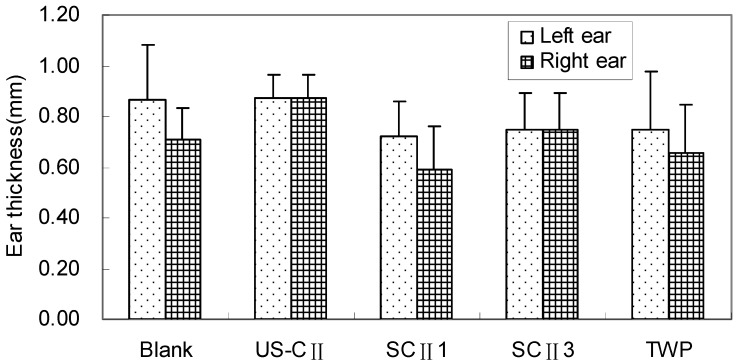
Effect of CII on DTH response in rats after immunization with CII and TWP: SCII was injected into the left ear intradermally on Day 7 and ear thickness measured 48 h later. The difference thickness between left and right ear in blank group, US-CII group, SCII 1 group and SCII 3 group, TWP group were 0.16 mm, 0.00 mm, 0.13 mm, 0.00 mm, and 0.10 mm, respectively.

To determine the effect of CII and TWP on the DTH response in the auto-immune-prone rat, SCII was injected into the left ear intradermally on Day 7 after immunization and the ear thickness was measured 48 h thereafter. As negative control, acetic acid (HAc) of 0.01 M concentration was injected into the right ear. Left ear thickness in CFA groups after injection with SCII was compared with the HAc-injected right ears (no SCII injected) thickness. As shown in Figure 3, the thickness of left ear and the thickness of right ear was almost the same in US-CII group and SCII 3 group. But in blank group, SCII 1 group and TWP group, left ear was thicker than right ear. It was calculated that the difference thickness between left and right ear in blank group, US-CII group, SCII 1 group, SCII 3 and TWP group were 0.16 mm, 0.00 mm, 0.13 mm, 0.00 mm, and 0.10 mm, respectively. This indicated that SCII, especially SCII 3, had a decreased DTH response in rats.

### 2.5. IL-10 Level in Serum

**Figure 4:**
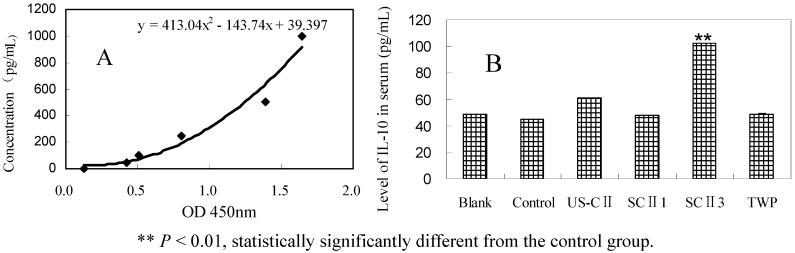
Effects of drugs on the expression of IL-10 in CFA rats (n = 10). (**A**) standard curve for the relationship between the concentrations of IL-10 and the absorbance at 450 nm; (**B**) the average level of IL-10 in serum of each experiment group.

After administration with different drugs for 2 weeks, the concentration of IL-10 in serum was measured by the method of ELISA. The concentrations of IL-10 in SCII, US-CII and TWP group were higher than that of control group (Figure 4). It was found that the release of IL-10 was induced by CII and TWP, especially by SCII 3 with significantly higher content of IL-10 102 pg/mL.

### 2.7. Histopathology of Arthritis

**Figure 5:**
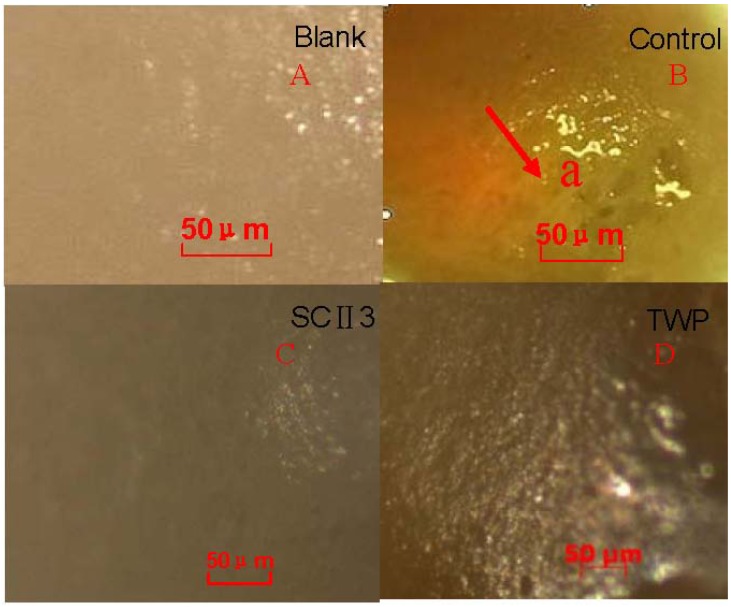
Photos of the articular cartilage surface. (**A**) the articular cartilage surface from rat in blank group; (**B**) the articular cartilage surface from arthritic rat in control group; (**C**) the articular cartilage surface from rat in SCII 3 group; (**D**) the articular cartilage surface from rat in TWP group. (**a**) Synovial membrane destruction.

To observe the effects of oral administrating SCII on the smoothness of the articular cartilage surface, rats were sacrificed after drugs administration for 2 weeks. The left ankles of blank group, control group, SCII 3 group, and TWP group were separated and stored in formaldehyde solution. Figure 5 illustrated the histopathological images of each group under the stereomicroscope. From the figure, it can be seen that surface of articular cartilage in the control group was apparently uneven. The articular cartilage surface in SCII 3 group and TWP group are relatively smooth comparing to the blank group.

### 2.8. Fas/Apo-1 Characteristic in T Cell Culture with US-CII and SCII

**Figure 6:**
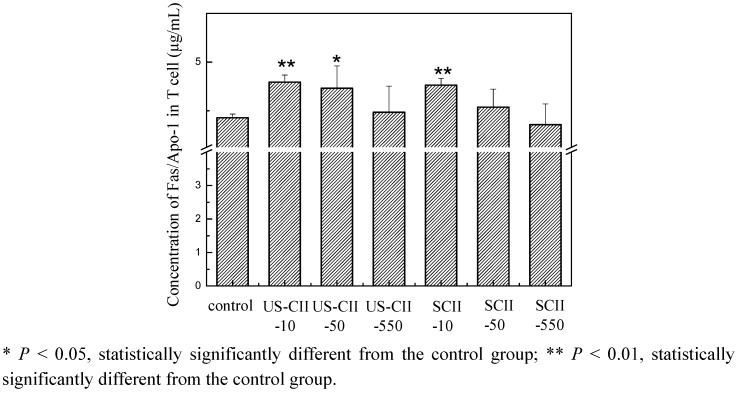
Effects of different CII on the concentration of Fas/Apo-1: The level of Fas/Apo-1 in the medium was measured by ELISA according to protocols of Human Fas/APO-1 ELISA kits.

Based on the above results, we further studied the mechanism of oral tolerance of SCII in T cells *in vitro*. The level of Fas/Apo-1 in the medium was measured by ELISA according to protocols of Human Fas/APO-1 ELISA kits. From Figure 6, we can see that, comparing to the control group with the concentration of Fas/Apo-1, US-CII group and SCII group with their concentration of 10 μg/L can significantly increase the level of Fas/Apo-1, which suggested that US-CII and SCII could help to enhance apoptosis of T cell.

## 3. Experimental

### 3.1. Experimental Animals

Wistar rats (male, 125–150 g, 3-week-old, n = 60) were purchased from Sino-British Sippr/BK Lab. Animal Ltd., Co., Shanghai, China. All rats were housed under standard laboratory conditions at 25 °C. During the experimental period, distilled water and commercially available food (from Sino-British Sippr/BK Lab Animal Ltd., Co.) were given freely and kept on sawdust in plastic-bottomed cages in groups. The lighting duration in the breeding room was 12 h (7:00 am to 7:00 pm). All experiments were approved by the Ethics Review Committee for Animal Experimentation of Marine Biopharmacy Department, Shanghai Ocean University.

### 3.2. Reagents

Blue shark (*Prionace glauca*) type II collagen was obtained from the Shanghai Institute of Fisheries Science. Firstly, to prepare shark cartilages for collagen extraction, the residual meat on the cartilage was removed manually and then cut into small pieces with the length of 1–2 cm. Extraction of collagen from shark cartilage was performed according to the methods of Kittiphattanabawon *et al*. [17] and Nalinanon *et al*. [18] with slight modifications. To remove non-collagenous protein, the prepared shark cartilage was treated with 0.1 mol/L NaOH and 0.5 mol/L ethylenediaminetetraacetic acid (EDTA) (pH 7.4). Acid soluble collagen was extracted from the pretreated cartilage by soaked in 0.5 mol/L acetic acid and precipitated by adding NaCl to final concentration of 2.6 mol/L in the presence of 0.05 mol/L Tris(hydroxymethyl) aminomethane, pH 7.5. Then the collagen was collected by centrifugation. The pellet was dissolved in 0.5 mol/L acetic acid and twice dialyzed against 25 volumes of 0.1 mol/L acetic acid. Next, the resulting dialysate was freeze-dried as powder. Type II collagen powder was dissolved in MilliQ purified water in a 1:10 w/v ratio. Thermolysin (55 U/mg) was added into the extracted collagen with the ratio of 1:100 w/w. Hydrolysis was performed in a shaker incubator for 2 h at 37 °C with agitation at 150 rpm. Thermolysin was inactivated at 99 °C for 10 min after hydrolysis. A supernatant was obtained by centrifugation at 15,700 × g for 10 min. Fraction was obtained by ultrafiltration of the hydrolyzate supernatants using 15-kDa and 10-kDa molecular weight cut off membranes. The ultrafiltrate was freeze-dried, vacuum packed and stored at −20 °C until further use. US-CII was purchased from Sigma Chemical Co. (St. Louis, MO, USA). TWP was made from Shanghai Sine Pharmaceutical Co., Ltd. Complete Freund’s Adjuvant (CFA) was purchased from Sigma Chemical Co. (St. Louis, MO, USA).

### 3.3. SDS-Polyacrylamide Gel Electrophoresis (SDS-PAGE) for SCII

SDS-PAGE was performed on gradient separating gels of 10% polyacrylamide using a 3% stacking gel as the method of Laemmli [19], but with slight modification. Collagen samples were mixed with 0.01 M Tris-HCl buffer (pH 8.0) containing 1% SDS and 1% 2-mercaptoethanol, 40% sucrose, 20% glycerol and 0.02% bromophenol blue, then electrophoresed at 25 A in vertical slab gels. Samples of 10 μg of collagen were loaded onto each gel. Gels were stained for 2 h in 1% Coomassie Brilliant Blue R-250 in methanol/acetic acid/water 5:2:5 (v/v/v) and distained in 5% methanol/7.5% acetic acid. The molecular weight of the collagen was estimated using a middle molecular weight calibration kit as marker.

### 3.4. Induction of CFA and Clinical Assessment of Arthritis

Experimental arthritis was induced in rats (n = 50) according to the method proposed by Newbould with some modifications [20,21,22]. The left footpad of each rat was injected with 0.1 mL of CFA with *Mycobacterium butyricum* 1% suspension in mineral oil (Sigma Chemical Co.). The development of RA disease was supervised daily by two observers. To quantitatively evaluate the severity of the RA, we used a scoring system that correlated the arthritis severity with joint size. Rat paws were scored for arthritis, as previously described [23]. Inflammation of the four pads was graded from 0 to 4: grade 0, pad with no swelling and focal redness; grade 1, pad with swelling of finger joints; grade 2, pad with mild swelling of ankle or wrist joints; grade 3, pad with severe inflammation; and grade 4, pad with deformity or ankylosis. Each paw was graded, so the maximum score was 16 for a rat. Meanwhile, two weeks after injecting, the joint thickness was measured by a slide caliper calibrated 0.01-mm graduations (Youfound Precision Co., Ltd., Hangzhou, China).

### 3.5. Administration of Type II Collagen

SCII was dissolved in 0.05 M acetic acid at 1 mg/mL (SCII 1) and 3 mg/mL (SCII 3). US-CII was dissolved in 0.05 M acetic acid at 1 mg/mL. TWP was dissolved in 0.05 M acetic acid at 10 mg/mL. 0.05 M acetic acid was prepared as blank. All of above prepared solutions were saved at 4 °C. Two weeks after treated by CFA, each rat in the drug test groups (n = 10 per group), according to its own weight, was administered the prepared SCII 1, SCII 3, US-CII and TWP respectively at an oral dose between 0.12 mL and 0.34 mL per day for 2 weeks. The control group (n = 10), according to its own weight, was given the prepared acetic acid at an oral of dose between 0.11 mL and 0.38 mL per day for 2 weeks. The normal rats (n = 10) without injecting CFA kept regular diet until the experiment ended.

### 3.6. Cell-Mediated Immunity to Type II Collagen

Cell-mediated immunity to type II collagen was determined by measurement of delayed type hypersensitivity (DTH) ear swelling on Day 7 after immunization, by a modification of the previously described technique [24]. Briefly, 20 μg (50 μL) of SCII in 0.01 M acetic acid was injected into the left ear intradermally in all rats and acetic acid alone was injected into the right ear at the same time. Prior to injection both ears were measured and the injection point marked to allow for injection at the same level as contra lateral ear. Ear thickness was measured after 48 h using slide caliper calibrated 0.01-mm graduations. 

### 3.7. Detection of IL-10 Production by ELISA

Two weeks after drug administration, the peripheral blood of rat was collected in 3.8% sodium citrate with the volume ratio of 10:1. The blood was centrifuged at 10,000 rpm for 10 min in a high speed tabletop centrifuge (TGL-16G, Shanghai Anting Scientific Instrument Factory, Shanghai, China). All the above serums were saved at −80 °C. Level of IL-10 in the serum was measured by ELISA according to protocols of the Rat Interleukin 10 ELISA kits (Shanghai BlueGene Biotech Co., Ltd., Shanghai, China).

### 3.8. Histomorphometry

The rats were killed by cervical dislocation. The left knee joint was dissected and fixed in buffered 10% formaldehyde at room temperature. The smoothness of the articular cartilage surface was observed under the SteREO Discovery. V12 (Carl Zeiss Microlmaging Co., Ltd., Shanghai, China).

### 3.9. Measurement of Tolerance in T cell induced by US-CII and SCII

Tolerance induced by US-CII and SCII was estimated by the level of Fas/Apo-1 in T cell. Two kinds of cell were used including rat CD4 T cells (Cat. No. 3H-2520-5, 3H Biomedical Co., Uppsala, Sweden) and rat T cells from the spleen of health Wister rat. The method of obtaining rat T cells was as follows: spleens were removed from rats and washed twice with PBS. Tissues were grinded and the cells were filtered through a cell strainer and centrifuged at 1,500 rpm for 5 min. There were two groups for the experiment: one is reference group, the other one is control group. In control group, cells (1 × 10^6^ cell/mL) contained rat T cells and rat CD4 T cells. Besides, rat T cells were treated with mytomycine C (50 μg/mL) at 37 °C for 30 min. In reference group, Cells (1 × 10^6^ cell/mL) only contained rat CD4 T cells. Both of these two groups were resuspended with various concentration of SCII (10 μg/L, 50 μg/L and 500 μg/L) or US-CII (10 μg/L, 50 μg/L and 500 μg/L). All kinds of cells were cultured in RPMI-1640 medium with 10% Fetal Bovine Serum at a concentration of 1 × 10^6^ cells/mL. The cells were cultured for 96 h at 37 °C in 5% CO_2_. Then the level of Fas/Apo-1 in the medium was measured by ELISA according to protocols of Human Fas/APO-1 ELISA kits.

### 3.10. Statistical Analysis

The data were presented as means ± standard deviation of three to ten determinations. Statistical analyses were performed using Student’s *t*-test and one-way analysis of variance. Multiple comparisons of means were done by LSD test. A probability value of <0.05 was considered significant. All computations were made by Origin 7.5 (Origin Lab Corp., Northampton, MA, USA).

## 4. Discussion

Oral administration of low doses of antigen induces a form of tolerance that can be transferred by T cells and potentiated by several agents [25]. Oral doses of the CII of bovine [26], chicken [12] and sheep [27], suppress experimental arthritis in various animal models through the responses of regulatory cells that mediate bystander suppression [28]. Oral tolerance can be assessed in many indexes of histomorphology, hematology and pathology [10,12,24]. In the present study, we demonstrated that immune tolerance was induced by shaker CII and the rheumatoid arthritis induced by complete freund’s adjuvant was reduced and repaired by the oral administration of shaker CII in rats from experimental evidences of histomorphology and clinical signs. 

Immune responses elicited by fed antigens differ from responses activated at other sites [3]. Typically, antigen feeding decreases delayed type hypersensitivity (DTH) responses and T response after immunization with the same antigen [28]. In this study, 3 mg kg^−1^d^−1^ SCII-fed rats showed reduced DTH significantly, which is a clear marker of the induction of systemic tolerance, while 1 mg kg^−1^d^−1^ SCII orally administrating rats did not show significant effect, suggesting that the appropriate amount of antigen is important for inducing tolerance. 

Appropriate dose of antigen ingestion favors active cellular suppression. One of the primary mechanism of active cellular suppression is the secretion of down regulating or suppressive cytokines such as TGF-β, IL-4 and IL-10 [8]. Compounds with modulatory properties might influence the tolerance induction in the mucosa [29]. For instance, the delivery of soluble peptides adsorbed to chitosan increases antigen release and maintains a sustained production of IL-10, facilitating tolerance induction [30]. Although the tolerance of SCII is characterized poorly, our data indicated the enhanced secretion of IL-10 upon the SCII feeding, suggesting that SCII might induce active cellular suppression. 

The T cell response generated in *vivo* depends upon the dose of the antigen, the route of delivery, the cytokine environment and the characteristics of antigen-presenting cells. Mechanisms of oral tolerance include anergy and/or active cellular suppression mediated by regulatory T cells [31]. In the present study, the stimulated increase of Fas/APO-1 in T cell by SCII indicated that this exogenous protein is capable of inducing antigen-specific immune tolerance. The T cell apoptosis of peripheral blood induced by SCII may result in the depletion of antigen-specific reactive T cells, which is also vital in inducing specific immune tolerance.

## 5. Conclusions

In conclusion, oral SCII can induce the specific suppression of cellular and humoral immune response in CFA-induced-rheumatoid-arthritis rats. The study enabled a better understanding of the mechanisms of oral tolerance and the regulation of cellular and humoral immune, which will help the development of innovative therapeutic intervention for RA.
